# Timing of first abdominal operation in Crohn’s disease based on a diagnostic model

**DOI:** 10.1038/s41598-024-55221-3

**Published:** 2024-03-13

**Authors:** Lichao Yang, Yawei Zhang, Baojia Yao, Qiang Wu, Liangxin Peng, Lianwen Yuan

**Affiliations:** Department of General Surgery, The Second Xiangya Hospital of Central South University, Changsha, 410011 China

**Keywords:** Crohn disease, Surgery, Nomogram, Inflammatory bowel disease, Gastroenterology, Gastrointestinal diseases, Endocrine system and metabolic diseases

## Abstract

This study aims to develop a clinical diagnostic model for assessing the need for initial abdominal surgery in patients diagnosed with Crohn's disease (CD) and create a nomogram to facilitate clinical decision-making. A total of 164 surgical CD patients and 230 control CD patients were included in this retrospective analysis. Least Absolute Shrinkage and Selection Operator (Lasso) regression and binomial logistic regression were employed to select clinical variables. The 394 CD patients were randomly allocated to a training set and a validation set in a 7:3 ratio. The filtered variables were used to establish a diagnostic model and nomogram in the training set, subsequently validated in the testing set. Decision Curve Analysis (DCA) and clinical impact curve were constructed to validate the clinical applicability of the model. Binomial logistic regression analysis identified seven clinical variables with a p-value less than 0.01, including Biomarker (B), Waist-to-Height Ratio (WHtR), Intestinal obstruction, Albumin (ALB), Crohn's Disease Activity Index (CDAI), Myocardial Flow Index (MFI), and C-reactive protein (CRP). These variables were utilized to establish the diagnostic model. Calibration curves showed good alignment, with a C-index of 0.996 in the training set and 0.990 in the testing set. DCA and clinical impact curve demonstrated that the diagnostic model had good clinical efficiency and could provide clinical benefits. A validated diagnostic model for determining the timing of the first abdominal operation in CD patients was established and evaluated, showing high discriminative ability, calibration, and clinical efficiency. It can be utilized by clinicians to assess the optimal timing for transitioning CD patients from medical treatment to surgical intervention, providing valuable references for individualized treatment decisions for CD patients.

## Introduction

Crohn’s disease (CD) is a type of inflammatory bowel disease which can affect any part from the mouth to the anus and it is also associated with extraintestinal manifestations^1^. The disease is believed to be caused by a combination of genetic, environmental, dietary, immune, and gut microbiota factors^2,3^. In recent years, the global incidence of CD has been increasing due to changes in diets, environments, and lifestyle habits, posing a significant threat to both the physical and mental health of individuals and contributing to the economic burden on patients and society as a whole^4^.

While medical treatment is the primary approach for managing CD, surgical intervention is often necessary when medical treatments are ineffective^5,6^. Research indicates that approximately 70% of CD patients require at least one surgical procedure in their lifetime, and the timing of surgical intervention can significantly impact clinical outcomes, particularly for initial abdominal surgery^6^. In clinical practice, the clinical symptoms of CD patients can be misleading, leading to missed opportunities for timely surgery, increased surgical risks, and higher chances of postoperative recurrence, while diminishing the benefits of surgery^5,7^. Therefore, this paper retrospectively analyzes the clinical characteristics and laboratory examination of CD patients who need to undergo the first abdominal operation, and establishes a predictive model to determine when CD patients will shift from medical treatment to surgical intervention, and to guide CD clinical decision-making.

## Materials and methods

### Patient identification

**Figure 1:**
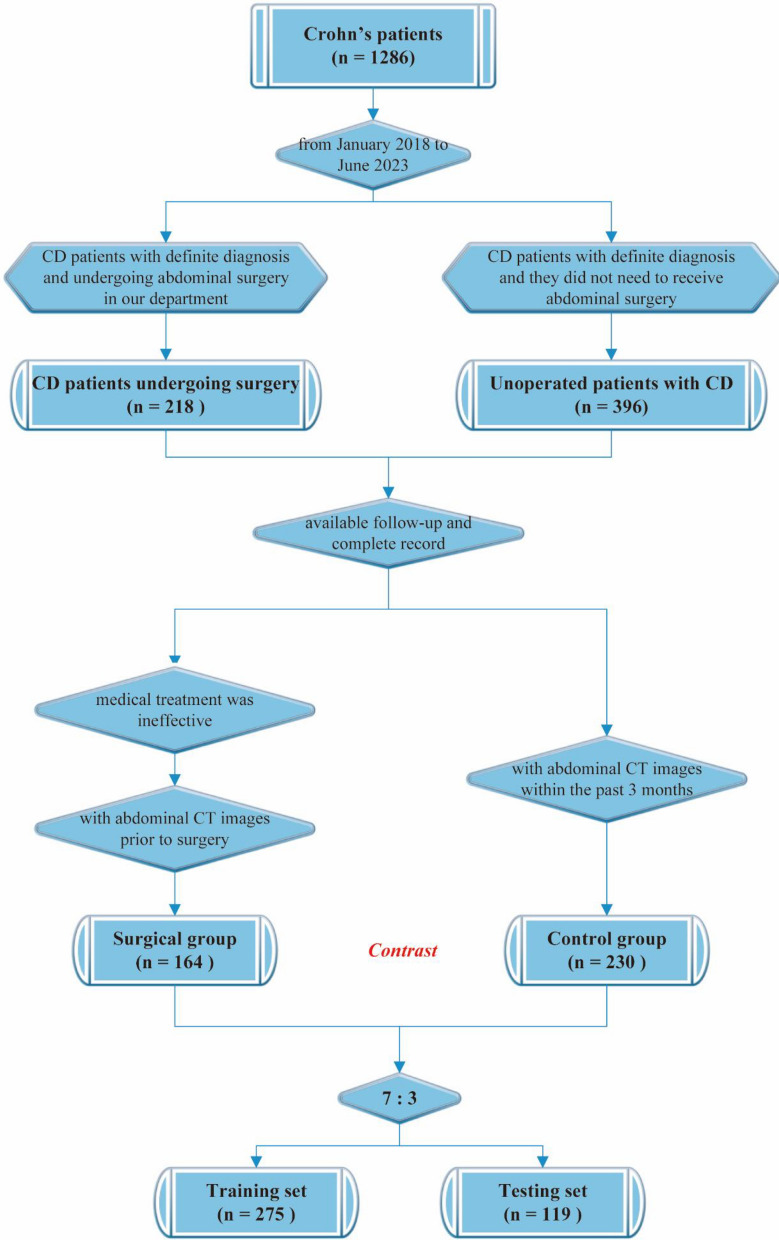
The flow chart of this study, including experimental grouping design and research process.

This study is a retrospective study. A clinical database for Crohn's disease patients has been established at The Second Xiangya Hospital of Central South University, encompassing 1286 individuals diagnosed with Crohn's disease who received treatment at our hospital from 2000 to 2023. Based on the inclusion and exclusion criteria, this study selected 164 CD patients who underwent initial intestinal resection surgery because of the medical treatment was ineffective at our hospital as the surgical group from the database (Fig. 1). Additionally, 230 CD patients who did not require surgical intervention were selected as the control group. In this study, we collected and analyzed patients' clinical information, including CT images, gender, age, disease type, personal history, clinical manifestations, disease behavior, disease location, and prognosis. The study was conducted in accordance with the guidelines of the Declaration of Helsinki and received approval from the Ethics Committee of The Second Xiangya Hospital of Central South University, Approval No. 2022–155. Informed consent and consent for publication were obtained from all subjects involved in the study.

### Inclusion and exclusion criteria

Patients who met the diagnostic criteria of the Chinese consensus, based on clinical, endoscopic, imaging, and pathological findings, were enrolled^8^. The inclusion criteria for the surgical group were as follows: (i) individuals who underwent their first abdominal operation at our hospital from January 2018 to June 2023 due to the ineffectiveness of medical treatment, and (ii) patients who had an abdominal CT examination within 1 week before the surgical intervention. For the control group, the inclusion criteria were: (i) individuals who had never undergone abdominal surgery from January 2018 to June 2023, (ii) patients who had an abdominal CT examination within the past 3 months, and (iii) those who demonstrated a positive response to medical treatment. Patients with incomplete medical information were excluded from both the surgical and control groups.

### Clinical variables

Demographic variables, such as age, gender, and smoking history, were included in the study. Clinical variables encompassed biochemical results at admission, symptoms, and the Montreal classification of Crohn's disease (CD)^9^. The Montreal classification, which provides a retrospective classification of CD lesion sites and phenotypes, was employed^9,10^. Given the challenge in distinguishing between the terminal ileum and jejunum, patients with lesions solely affecting the small intestine were classified as L1 type.

### Surgical indications

The main indications for surgery in Crohn's disease (CD) included perforation, formation of internal or external fistulas, abscess formation, intestinal obstruction, refractory bleeding, and ineffective medical treatment^11–13^. The choice of surgery for patients was decided through discussion at the multidisciplinary team (MDT) conference, including gastroenterologists, gastrointestinal surgeons (geriatric surgeons), pathologists, radiologists, and non-core members, including nutritionists, psychiatrists, and nurses^14^. The MDT conference on patients with CD was held once a week. For this study, only patients who underwent surgical intervention due to the inefficacy of medical treatment were selected.

### MFI measurement

The patients underwent supine abdominal CT scans, and CT images were taken at the level of the umbilicus (L3 or L4). The Hounsfield unit values of adipose tissue ranged from -190 HU to -30 HU^15^. The Magic Wand tool in Adobe Photoshop CS3 was used to trace the areas of subcutaneous and visceral adipose tissue, and their areas were measured in square centimeters based on pixel count. The mesenteric fat index (MFI) was calculated for each patient as the ratio of the visceral fat area to the subcutaneous fat area^15–17^.

### LAP and WHtR measurement

Waist circumference (WC) refers to the horizontal circumference of the waist through the umbilical point^18^. The Lipid accumulation product (LAP) of Male was calculated using the formula [WC (cm)—65] × TG (Triglyceride, mmol/L), while female LAP was calculated using the formula [WC (cm)—58] × TG (mmol/L)^19^. The waist-to-height ratio (WHtR) was defined as the ratio of waist circumference (cm) to height (cm)^18,20^.

### Statistical analysis

All statistical analyses and displays were conducted using R software version 4.2.0. The R code can be found in the supplementary document. Descriptive statistics, including the median and interquartile range for continuous variables, and frequencies for categorical variables, were calculated for all variables. The discrimination performance of the nomogram prediction model was assessed using the area under the receiver-operating characteristic curve (AU-ROC) and the consistency index (C-index). Calibration curves were plotted to evaluate the calibration of the nomogram prediction model. Decision curve analysis (DCA) and clinical impact curve were performed to determine the clinical usefulness of the nomogram. DCA compared the net benefits of each prediction model at different threshold probabilities. The net benefit was calculated by subtracting the proportion of false-positive patients from the proportion of true-positive patients, considering the relative harm of forgoing interventions compared to the negative consequences of unnecessary interventions. All reported statistical significance levels were 2-sided, with a significance threshold set at 0.05. All figures were generated using Adobe Illustrator software (version 2022).

## Result

### Baseline characteristics of the participants

A total of 394 patients with Crohn's disease (CD) were included in this study, with 164 patients in the surgical group and 230 patients in the control group. The median age of patients in the surgical group was 29 years (interquartile range: 22.85–38.12), while the median age of patients in the control group was 27 years (interquartile range: 21.00–33.00). The difference in age between the two groups was statistically significant (p < 0.01). The male-to-female ratio was approximately 3:1 in both groups (p > 0.05), but the proportion of smokers in the surgical group was approximately twice that of the control group (p < 0.01). The primary clinical symptom for both groups was abdominal pain, but the proportion of individuals with abdominal pain was significantly higher in the surgical group compared to the control group (p < 0.01). Furthermore, the number of patients with symptoms of intestinal obstruction was significantly lower in the control group than in the surgical group (p < 0.01). The Montreal classification, laboratory test results, and other clinical variables of these 394 CD patients were summarized in Supplementary Table 1.

### Selection of clinical variables

**Figure 2:**
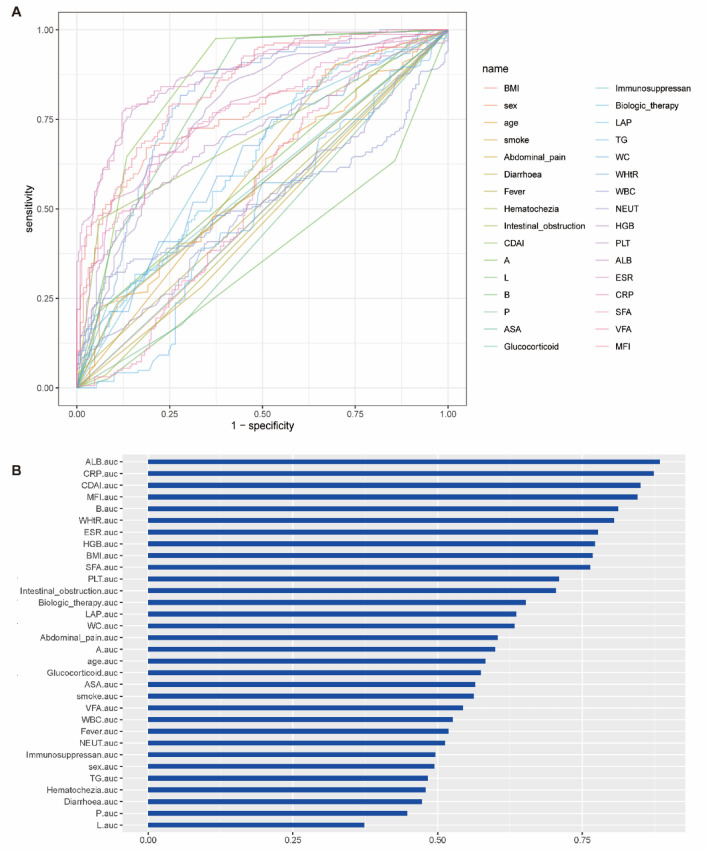
The Receiver Operating Characteristic (ROC) curve of all the clinical variables. (**A**) ROC curve. (**B**) AUC value of all the clinical variables.

**Figure 3:**
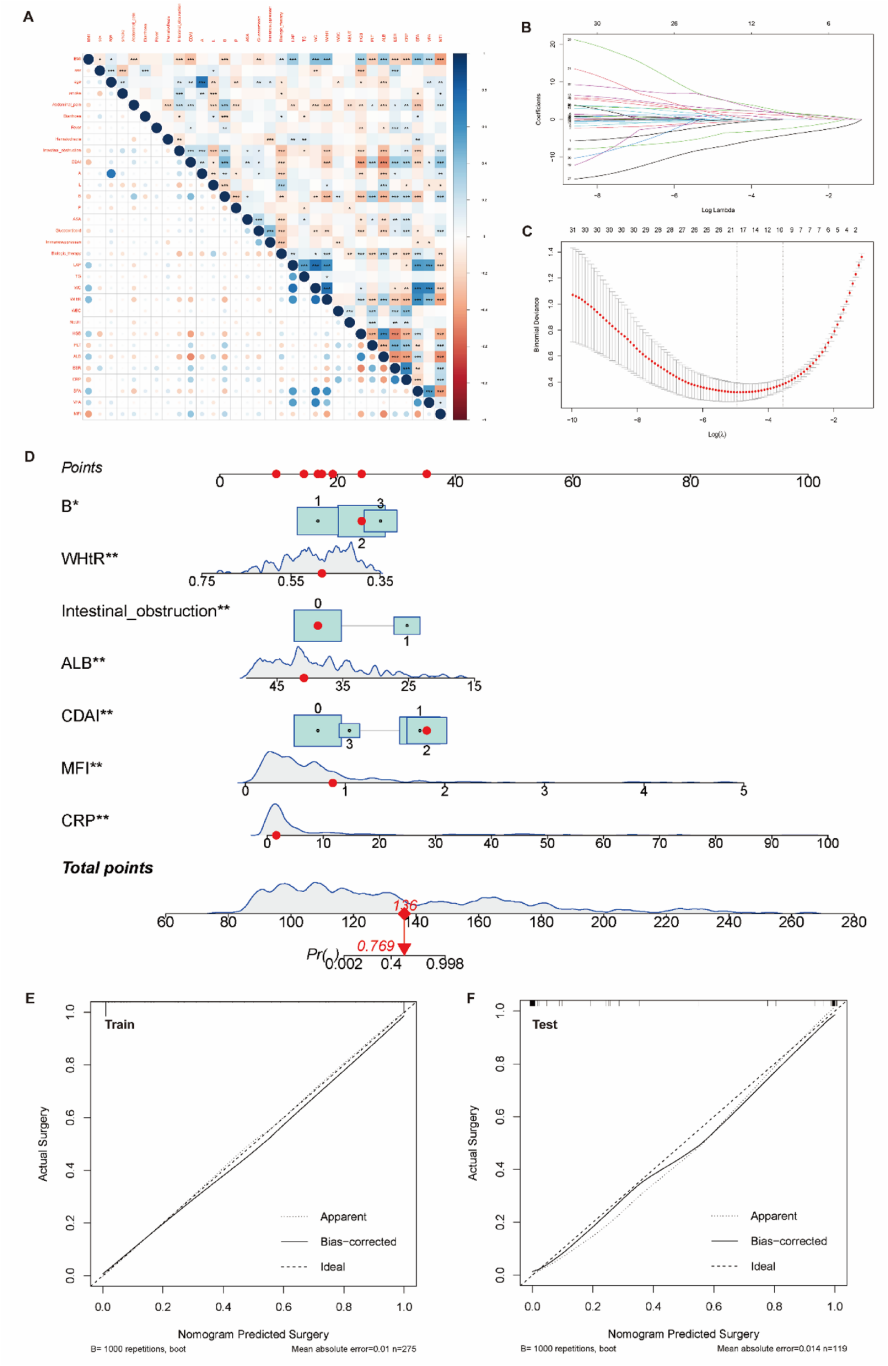
Screening of clinical variables and establishment of diagnostic model. (**A**) Correlation analysis of all clinical variables (*p < 0.05, **p < 0.01, ***p < 0.001). (**B**) LASSO coefficient profiles of all clinical variables. (**C**) Cross validation plot for the penalty term, and the results showed that 18 variables were retained when the error was the smallest; that is, the place corresponding to the dotted line on the left. To avoid overfitting and simplicity of the model, no more than one standard error was selected compared with the minimum error, and 9 variables were retained, which corresponded to the place on the dotted line on the right. (**D**) The 9 variables screened by Lasso Regression were analyzed by Binomial Logistic Regression Analysis again, and only 7 variables with p value less than 0.01 were used to establish the diagnostic model and nomogram. (**E**) Calibration curve of the model in the training set. (**F**) Calibration curve of the model in the testing set.

In this study, the dependent variable was the need for abdominal intestinal resection surgery, while the independent variables consisted of 32 clinical variables. Receiver operating characteristic (ROC) analysis was performed on these 32 clinical variables (Fig. 2), and the variable with the highest area under the curve (AUC) value was albumin (ALB). Considering the presence of correlations among these 32 variables (Fig. 3A), lasso regression was conducted to address issues of multicollinearity and overfitting (Fig. 3B and C). From the lasso regression, a total of 9 variables were selected for further analysis. To further reduce the number of variables and enhance the accuracy of the model, the 9 variables selected from the lasso regression were subjected to binomial logistic regression analysis, which revealed that 7 variables exhibited statistical significance (Table 1).

**Table 1:** The variables screened by Lasso Regression were analyzed by Binomial Logistic Regression Analysis again.

	Estimate	Std. error	z value	Pr ( >|z|)
(Intercept)	9.745814768	3.334552311	2.922675627	3.47E − 03
MFI	2.700780226	0.9165333	2.946734426	3.21E − 03
CRP	0.179456976	0.061537528	2.916220126	3.54E − 03
ALB	− 0.29368496	0.065765087	− 4.46566667	7.98E − 06
PLT	0.003222585	0.002864874	1.124860969	2.61E − 01
Intestinal obstruction	2.68042496	0.675089609	3.970472844	7.17E − 05
WHtR	− 12.8081591	4.69207161	− 2.72974502	6.34E − 03
B	2.210546402	0.509488494	4.338756279	1.43E − 05
CDAI	1.124518572	0.333399539	3.372885808	7.44E − 04
BMI	− 0.10841952	0.090984228	− 1.19162979	2.33E − 01

*MFI* Mesenteric fat index, *CRP* C-reactive protein, *ALB* Albumin, *PLT* Platelet count, *WHtR* Waist-to-height ratio, *B* Behavior of disease, *CDAI* Crohn's Disease Activity Index, *BMI* Body mass index.

### Development and validation of the diagnostic model and nomogram

**Figure 4:**
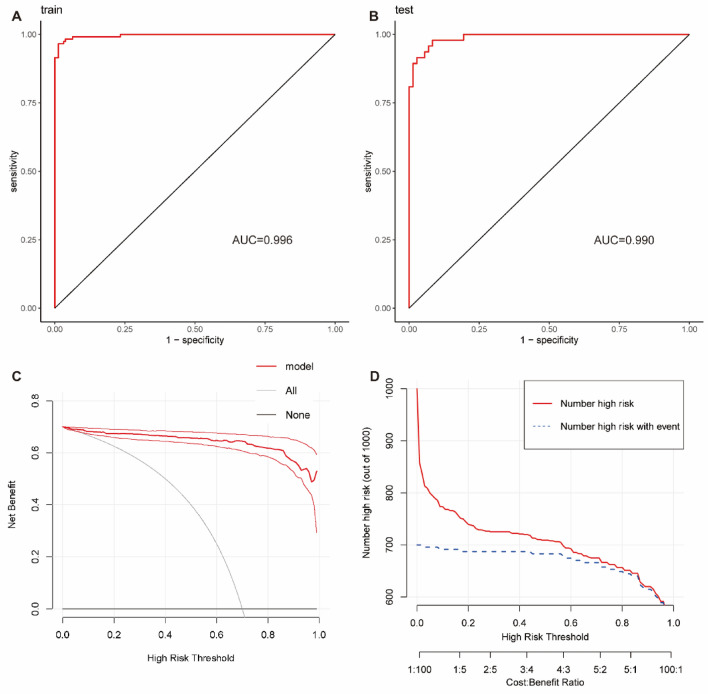
Diagnostic efficacy and clinical application value of the model. (**A**) ROC curve of the model in the training set. (**B**) ROC curve of the model in the testing set. (**C**) The decision curve analysis of the nomogram prediction model, and the light red curve range is 95% confidence interval; With the change of threshold probability, the net benefit of intervention according to the predicted value of the model was higher than that of All intervention or None intervention. (**D**) The clinical impact curve of the nomogram prediction model, the red solid curve represented the number of patients classified as need to receive surgery in CD by this nomogram under each risk threshold of 1000 patients, and the blue dashed curve showed the number of true CD patients under each risk threshold.

The binomial logistic regression analysis identified seven clinical variables with a p-value less than 0.01, which included B, WHtR, Intestinal obstruction, ALB, CDAI, MFI, and CRP. These seven variables were utilized to establish the diagnostic model and create a nomogram in the training set (Fig. 3D). The nomogram in Table 2 illustrates the proportions and statistical significance of these variables. The density plot of total points and age depicts their distribution. For categorical variables, their distributions are represented by the size of the box. The importance of each variable was ranked based on the standard deviation along the nomogram scales. To utilize the nomogram, individual patients' specific points (indicated by red dots) are located on each variable axis. Red lines and dots are then drawn upwards to determine the points received for each variable. The sum of these points is located on the Total Points axis, and a line is drawn downward to determine the probability of CD patients requiring initial abdominal surgery. The calibration curves of the model generated in the training set and the test set demonstrate that the straight line and the corrected straight line deviate only slightly from the ideal straight line, indicating good diagnostic consistency and high calibration (Fig. 3E and F). The C-index for the prediction nomogram was 0.996 for the training set and 0.990 for the testing set, respectively (Fig. 4A and B).

**Table 2:** The proportion and statistical significance of variables in the nomogram.

	Coef	S.E	Wald Z	Pr ( >|Z|)
Intercept	16.5247	7.6191	2.17	0.0301
MFI	6.5139	2.1076	3.09	0.0020
CRP	0.3664	0.1229	2.98	0.0029
ALB	− 0.4300	0.1396	− 3.08	0.0021
Intestinal obstruction = 1	5.8317	2.1729	2.68	0.0073
B = 2	2.864	1.4833	1.93	0.0535
B = 3	4.0973	1.7255	2.37	0.0176
WHtR	− 29.1292	11.1229	− 2.62	0.0088
CDAI = 1	6.6714	2.3711	2.81	0.0049
CDAI = 2	7.1184	2.5097	2.84	0.0046
CDAI = 3	2.0664	1.8836	1.1	0.2726

*B* Behavior of disease (2: Stricture, 3: fistula), *CDAI* Crohn's Disease Activity Index (1: Mild, 2: Moderate, 3: Severe).

### Clinical application value of the nomogram

The clinical utility of using decision curve analysis (DCA) to predict the need for surgical intervention in CD patients was assessed by evaluating the net benefits. The DCA demonstrated that, as the threshold probability varied, the net benefits of intervention based on the model's predicted values surpassed those of full intervention or no intervention, indicating that clinical interventions guided by this decision curve yielded greater net benefits (Fig. 4C). Additionally, clinical impact curves were generated to analyze the number of patients classified as high risk by the decision curve and the number of true positive patients at each risk threshold (Fig. 4D). To achieve a balance between high net benefits and low false-positive rates, DCA was combined with the clinical impact curve. The calibration indicated that setting the risk threshold for CD above 0.70 provided higher clinical benefits and lower false-positive rates for the entire population under consideration.

## Discussion

To date, the clinical treatment of Crohn's disease (CD) primarily relies on medication, including hormones, aminosalicylates, immunosuppressants, and biologics^2,4^. However, due to individual differences, CD patients exhibit varying sensitivities to medications^21^. While CD patients often receive multiple drug therapies simultaneously, medication treatment has drawbacks such as significant side effects, poor drug tolerance, limited long-term maintenance, and high cost^21,22^. Consequently, for CD patients who do not respond well to medication treatment, the decision of whether to continue switching medications or opt for surgical intervention poses a major challenge in the clinical treatment of CD.

CD is a debilitating disease characterized by chronic inflammation in the intestines, often leading to malnutrition and weight loss in patients. Some patients may experience decreased physical strength and an inability to tolerate daily activities^2,3,8^. For CD patients who struggle to maintain remission, frequent medication changes may further deplete their body reserves due to the side effects of multiple drugs, and they may miss the opportunity for timely surgical removal of intestinal inflammatory lesions. Therefore, actively choosing surgical intervention instead of waiting for medication treatment to fail can reduce the surgical risk for CD patients, improve their response to medication, and potentially achieve long-term disease remission or even lifelong remission^5^. Surgical indications of CD include unclear diagnosis, intestinal perforation (fistula), intestinal obstruction, malignancy, bleeding, impaired growth and development, among others. Patients may present with multiple surgical indications simultaneously. We have summarized the primary surgical indications for all CD surgical patients in Supplementary Table 2. Due to the complex and variable clinical symptoms of CD, surgical indications are challenging to determine. Therefore, we attempted to explore the potential connections between the clinical presentation of CD and the need for surgical intervention by summarizing and analyzing the clinical data of CD patients undergoing surgery, using statistical tools. Due to the fact that CD patients often initially seek consultation in the field of gastroenterology, undergo standardized medical treatment, and the determination of whether surgical intervention is necessary relies heavily on the subjective judgment of gastroenterologists, potentially associated with the clinical experience of the physician. Therefore, the development of a relatively objective CD surgical prediction model is helpful in guiding clinical treatment, facilitating the timely and efficient selection of the optimal timing for surgical intervention.

CD patients often exhibit systemic inflammation accompanied by disturbances in lipid metabolism^23^. Our previous research demonstrated a correlation between mean fat attenuation (MFI) values, used to evaluate abdominal fat distribution, and CD postoperative recurrence and anastomotic ulceration^24^. However, since MFI values only reflect local fat metabolism and do not effectively reflect systemic fat metabolism, this study also includes BMI, WC, WHtR, and LAP as four clinical variables that can assess overall body fat metabolism^18,20^. A multidimensional analysis from local to systemic aspects was conducted to identify differences between CD patients requiring surgery and the control group.

In this study, data collection for the surgical group was limited to 1 week before surgery, while data collection for the control group was restricted to the most recent hospitalization within the past 3 months. We successfully established a diagnostic model for determining the optimal timing for surgical intervention in CD patients. The model includes seven variables: B, WHtR, intestinal obstruction, ALB, CDAI, MFI, and CRP. Compared with type B1 CD patients, stenotic lesions and penetrating lesions are more likely to cause surgery. Patients with CD usually have mild or moderate CDAI during surgery, because surgical intervention is unnecessary when CDAI is in remission, while in severe active stage, patients may not be able to bear the risk of surgical intervention. CRP is an inflammatory factor, which is mainly used to reflect the level of inflammatory activity in vivo, and is positively correlated with surgical intervention. ALB and WHtR represent the overall nutritional status of the patients, and intestinal obstruction is defined as the presence of clinical symptoms or imaging findings suggestive of intestinal obstruction. Furthermore, the model demonstrated good internal validation, high calibration, and high clinical utility in our dataset. However, this is a single-center study with a limited number of patients, and all patients belong to the Chinese Han nationality. Our study may have ethnic and population biases, and therefore, further research with larger sample sizes from multiple centers is needed to validate this diagnostic model.

In summary, the diagnostic model for determining the optimal timing of surgical intervention in CD patients has significant clinical utility and can partially mitigate the subjective biases of clinicians relying solely on their clinical experience to make decisions regarding the transition from medical to surgical treatment for CD. As the treatment of CD is lifelong, choosing the best timing for abdominal operation when it becomes unavoidable can greatly benefit the patients.

## Conclusion

We identified seven clinical variables, including B, WHtR, intestinal obstruction, ALB, CDAI, MFI, and CRP, to construct a diagnostic model for determining the necessity of surgery in CD. This validated diagnostic model, aimed at determining the optimal timing for the first abdominal operation in CD patients, was established and evaluated. It demonstrated high discriminative ability, calibration, and clinical efficiency. Clinicians can utilize it to assess the optimal transition timing for CD patients from medical treatment to surgical intervention, providing valuable references for individualized treatment decisions in CD patients.

### Supplementary Information

Supplementary Tables.

## Data Availability

The datasets used and/or analysed during the current study available from the corresponding author on reasonable request.
