# Supplementary material for: Timing of first abdominal operation in Crohn’s disease based on a diagnostic model
Source: Sci Rep. 2024 Mar 13;14:6099. doi: 10.1038/s41598-024-55221-3 (PMC10937665; doi:10.1038/s41598-024-55221-3)
Supplement: Supplementary file 1 — Supplementary Tables. [file 41598_2024_55221_MOESM1_ESM.docx]

Supplementary table 1: Demographic and clinical characteristics of 394 Patients with Crohn's Disease (*p<0.05, **p<0.01, ***p<0.001).

| **Characteristics** | **Total (n = 394)** | **Control group (n = 230)** | **Surgical group (n = 164)** | **p-value** |
| --- | --- | --- | --- | --- |
| SFA, (cm2, Median, (Q1, Q3)) | 122.56 (68.2, 191.77) | 155.8 (105.17, 233.78) | 73.8 (38.38, 126) | *** |
| VFA, (cm2, Median, (Q1, Q3)) | 57.44 (36.42, 91.76) | 53.37 (32.41, 91.76) | 59.56 (42.72, 91.06) | 0.14 |
| MFI, (Median, (Q1, Q3)) | 0.51 (0.30, 0.85) | 0.37 (0.25, 0.54) | 0.82 (0.59, 1.3) | *** |
| WC, (cm, Median, (Q1, Q3)) | 75.43 (70.71, 81.14) | 77 (71.71, 82.71) | 72.29 (69.14, 77.39) | *** |
| WHtR, (Median, (Q1, Q3)) | 0.48 (0.43, 0.54) | 0.52 (0.47, 0.57) | 0.44 (0.42, 0.47) | *** |
| LAP, (cm·mmol/L, Median, (Q1, Q3)) | 14.97 (8.42, 22.49) | 17.15 (9.82, 27.4) | 12.47 (7.03, 18.48) | *** |
| BMI, (kg/m2, Median, (Q1, Q3)) | 19.41 (17.76, 22.19) | 20.45 (19.03, 23.86) | 17.77 (16.03, 19.67) | *** |
| age, (years, Median, (Q1, Q3)) | 28 (21.00, 36.00) | 27 (21.00, 33.00) | 29 (22.85, 38.12) | ** |
| sex, n (%) |  |  |  | 0.89 |
| Male | 293 (74) | 170 (74) | 123 (75) |  |
| Female | 101 (26) | 60 (26) | 41 (25) |  |
| Smoke history, n (%) |  |  |  | ** |
| No | 329 (84) | 204 (89) | 125 (76) |  |
| Yes | 65 (16) | 26 (11) | 39 (24) |  |
| Abdominal pain, n (%) |  |  |  | *** |
| No | 86 (22) | 70 (30) | 16 (10) |  |
| Yes | 308 (78) | 160 (70) | 148 (90) |  |
| Diarrhoea, n (%) |  |  |  | 0.3 |
| No | 271 (69) | 153 (67) | 118 (72) |  |
| Yes | 123 (31) | 77 (33) | 46 (28) |  |
| Fever, n (%) |  |  |  | ** |
| No | 388 (98) | 230 (100) | 158 (96) |  |
| Yes | 6 (2) | 0 (0) | 6 (4) |  |
| Hematochezia, n (%) |  |  |  | 0.13 |
| No | 370 (94) | 212 (92) | 158 (96) |  |
| Yes | 24 (6) | 18 (8) | 6 (4) |  |
| Intestinal obstruction, n (%) |  |  |  | *** |
| No | 303 (77) | 216 (94) | 87 (53) |  |
| Yes | 91 (23) | 14 (6) | 77 (47) |  |
| Disease Activity (CDAI, n (%)) |  |  |  | *** |
| Remission | 148 (38) | 144 (63) | 4 (2) |  |
| Mild | 109 (28) | 55 (24) | 54 (33) |  |
| Moderate | 106 (27) | 21 (9) | 85 (52) |  |
| Severe | 31 (8) | 10 (4) | 21 (13) |  |
| A classification at diagnosis, n (%) |  |  |  | *** |
| 1 | 32 (8) | 25 (11) | 7 (4) |  |
| 2 | 313 (79) | 191 (83) | 122 (74) |  |
| 3 | 49 (12) | 14 (6) | 35 (21) |  |
| L classification at diagnosis, n (%) |  |  |  | *** |
| 1 | 79 (20) | 28 (12) | 51 (31) |  |
| 2 | 14 (4) | 5 (2) | 9 (5) |  |
| 3 | 278 (71) | 178 (77) | 100 (61) |  |
| 4 | 23 (6) | 19 (8) | 4 (2) |  |
| B classification at diagnosis, n (%) |  |  |  | *** |
| 1 | 135 (34) | 131 (57) | 4 (2) |  |
| 2 | 174 (44) | 78 (34) | 96 (59) |  |
| 3 | 85 (22) | 21 (9) | 64 (39) |  |
| Perianal involvement at diagnosis, n (%) |  |  |  | * |
| No | 300 (76) | 165 (72) | 135 (82) |  |
| Yes | 94 (24) | 65 (28) | 29 (18) |  |
| ASA, n (%) |  |  |  | ** |
| No | 330 (84) | 205 (89) | 125 (76) |  |
| Yes | 64 (16) | 25 (11) | 39 (24) |  |
| Glucocorticoid, n (%) |  |  |  | *** |
| No | 337 (86) | 211 (92) | 126 (77) |  |
| Yes | 57 (14) | 19 (8) | 38 (23) |  |
| Immunosuppressan, n (%) |  |  |  | 0.95 |
| No | 272 (69) | 158 (69) | 114 (70) |  |
| Yes | 122 (31) | 72 (31) | 50 (30) |  |
| Biologic therapy, n (%) |  |  |  | *** |
| No | 211 (54) | 94 (41) | 117 (71) |  |
| Yes | 183 (46) | 136 (59) | 47 (29) |  |
| Results of laboratory examination | | | | |
| WBC, (10^9/L, Median, (Q1, Q3)) | 5.66 (4.49, 6.92) | 5.64 (4.63, 6.55) | 5.72 (4.28, 8.07) | 0.37 |
| NEUT, (%, Median, (Q1, Q3)) | 61.55 (55.03, 71.2) | 61.3 (55.9, 71.1) | 62.9 (53.9, 71.7) | 0.67 |
| HGB, (g/L, Median, (Q1, Q3)) | 133 (111.25, 144) | 140.5 (126, 149) | 116 (97, 133) | *** |
| PLT, (10^9/L, Median, (Q1, Q3)) | 255 (204, 320.5) | 237 (191.25, 275.75) | 303.5 (234.25, 383.75) | *** |
| ALB, (g/L, Median, (Q1, Q3)) | 40.25 (34.9, 44.38) | 42.75 (40.1, 46.4) | 34.45 (28.85, 38.12) | *** |
| ESR, (mm/hour, Median, (Q1, Q3)) | 11 (6, 22) | 7 (4, 13.75) | 21 (10, 36.25) | *** |
| CRP, (mg/L, Median, (Q1, Q3)) | 2.21 (1.07, 6.81) | 1.23 (0.89, 2.22) | 8.85 (3.78, 22.06) | *** |
| TG, (mmol/L, Median, (Q1, Q3)) | 1.24 (0.96, 1.75) | 1.21 (0.96, 2) | 1.24 (0.97, 1.57) | 0.55 |

Supplementary table 2: The surgical indications of the CD patients.

| **Surgical indications** | **Number of patients (N=164)** | **Ratio (%)** |
| --- | --- | --- |
| Undetermined Diagnosis | 30 | 18.29 |
| Intestinal Perforation (Intestinal Fistula/External Fistula) | 20 | 12.20 |
| Intestinal Obstruction | 34 | 20.73 |
| Intestinal Perforation Complicated by Intestinal Obstruction | 59 | 35.98 |
| Intestinal Bleeding | 2 | 1.22 |
| Suspected Malignancy | 3 | 1.83 |
| Impaired Growth and Development | 5 | 3.05 |
| Chronic Malnutrition | 11 | 6.71 |
